# Clinical Significance of Neutrophil Gelatinase-Associated Lipocalin in Crimean-Congo Hemorrhagic Fever

**DOI:** 10.1155/2015/374010

**Published:** 2015-01-22

**Authors:** Ayse Erturk, Erkan Cure, Emine Parlak, Medine Cumhur Cure, Serap Baydur Sahin, Suleyman Yuce

**Affiliations:** ^1^Department of Infectious Disease, School of Medicine, Recep Tayyip Erdogan University, Islampasa Mahallesi, 53100 Rize, Turkey; ^2^Department of Internal Medicine, School of Medicine, Recep Tayyip Erdogan University, Rize, Turkey; ^3^Department of Infectious Disease, School of Medicine, Ataturk University, Erzurum, Turkey; ^4^Department of Biochemistry, School of Medicine, Recep Tayyip Erdogan University, Rize, Turkey; ^5^Department of Endocrinology, School of Medicine, Recep Tayyip Erdogan University, Rize, Turkey

## Abstract

Neutrophil gelatinase-associated lipocalin (NGAL), which is an important prognostic marker for sepsis and inflammatory diseases, is mostly released from neutrophils. Crimean-Congo hemorrhagic fever (CCHF) patients are generally neutropenic. We aimed to investigate whether there is a change in serum NGAL level and to investigate its effect on the recovery time (RT) during the course of CCHF. A total of 40 CCHF patients (19 females and 21 males) and 34 healthy controls (17 females and 17 males) were included in the study. The serum NGAL level and biochemical and hematological parameters were checked. The NGAL level of CCHF patients was significantly higher than that of the healthy controls (*P* < 0.001). A multivariate analysis showed that the independent prognostic factor for the prediction of the RT is the NGAL level (odds ratio [OR] 0.3, 95% confidence interval [Cl] 0.1–0.4, *P* < 0.001). An elevated NGAL level was found to be associated with an increased RT in CCHF patients. The NGAL levels of CHHF patients might be elevated due to increased cytokine release, the presence of a tissue injury, and the release of immature neutrophils from the bone marrow into the peripheral stream. This may be a good prognostic factor in CHHF patients.

## 1. Introduction

Neutrophil gelatinase-associated lipocalin (NGAL) (lipocalin-2) is an endogenous bacteriostatic protein that is released from renal tubular epithelial cells, neutrophils, and macrophages [1]. NGAL is important as a host defense because it behaves as an antibacterial agent by scavenging bacterial siderophores to prevent bacteria from establishing an infection [2]. However, the positive association between tumor necrosis factor-alpha and the NGAL level is known [3]. In addition, it has been reported that there is a correlation between systemic inflammation and NGAL level [4], which has been reported to be a prognostic factor predicting the severity of acute renal failure [5] and septic infections [6]. In addition, an elevated NGAL level has been found to be able to predict severe infection courses [6, 7]; therefore, it may indirectly show increased inflammation.

Crimean-Congo hemorrhagic fever (CCHF) is a potentially fatal viral disease caused by a* Nairovirus*, a member of the Bunyaviridae family [8]. CCHF has been reported to be endemic in some regions of Turkey, especially in and around Erzurum and the Black Sea region during the summer months [9]. Its course has mostly mild clinical signs. However, sometimes it may be fatal. Many clinical conditions may be seen during CCHF, including organ failures, mainly of the liver and kidneys, as well as coagulopathy, hypofibrinogenemia, neutropenia, and thrombocytopenia [10]. During the course of the disease, hemophagocytosis occurs due to the intensive release of the cytokines, such as tumor necrosis factor-alpha, interleukin-6, interleukin-1, and interferon, which leads to neutropenia, lymphopenia, and thrombocytopenia [11, 12].

Contrary to aspartate aminotransferase (AST) and alanine aminotransferase (ALT), platelet count is a prognostic factor that demonstrates the progression of the disease [13]. However, a good marker is not available for a prognosis of the disease. CCHF is progressive with neutropenia. As NGAL is mainly released from neutrophils, its serum level is changeable in CCHF patients, which may show the severity of the disease. In this study, we aimed to investigate whether there is a relation between serum NGAL level and recovery time (RT) in CCHF patients, as well as whether its level is predictive for the RT of the disease.

## 2. Methods

### 2.1. Study Design

A total of 40 CCHF patients (19 females and 21 males) and 34 healthy controls (17 females and 17 males) were included in the study. The study was carried out in the Infectious Diseases Department of Ataturk University. This study was performed following the guidelines of the Helsinki Declaration and it was approved by the local ethics committees. Subjects who had certainly never been exposed to tick bites and who did not have any acute or chronic inflammatory diseases or infectious diseases were included in the control group. Patients with a history of diabetes, hypertension, hyperlipidemia, coronary artery disease, chronic obstructive pulmonary disease, cirrhosis, portal hypertension, hematological disorders, and malignancies were excluded from the control group. All the subjects were nonsmokers and were not consuming alcohol or using drugs.

#### 2.1.1. Selection of the Patients

The patients who were exposed to tick bites were followed up every day from the time of exposure and were called to policlinic control. During the followup, patients who showed the disease symptoms, such as high fever, chills, severe headache, dizziness, fatigue, myalgia, back and abdomen pain, nausea, vomiting, bloody diarrhea, or mucosal-skin hemorrhagic lesions, or those with impaired laboratory parameters were considered as having CCHF and were hospitalized to our ward. A blood sample of 1.5 mL was drawn from each patient and was sent according to the rules of cold chain to the Refik Saydam Hygiene Institute (CCHF reference center) in Ankara, Turkey, a CCHF reference laboratory. At this reference center, the diagnosis was confirmed by detecting the nucleic acids via real-time reverse transcriptase polymerase chain reaction. According to the polymerase chain reaction results, patients with positive viral RNA were confirmed as having CCHF and were included in the study [14]. The first day of the disease was accepted to be the day in which the polymerase chain reaction test was positive. The selected patients of our study were nonsevere cases, according to Swanepoel et al.'s fatality criteria, due to the low mortality rate of CCHF in our region [15].

#### 2.1.2. RT

When the patient's symptoms were decreased and the white blood cell, platelet, coagulation test, AST, ALT, urea, and creatinine levels were normal, samples for polymerase chain reaction were taken from the patients to ensure the disappearance of viral RNA, and the patients were considered to be completely recovered. The interval between the day in which the viral RNA was detected and the day of its disappearance was considered the RT.

### 2.2. Biochemical and Hematological Parameters

All biochemical parameters were measured after a fasting period of 12 h. Then, the serum samples were stored at −30°C. All the biochemical parameter levels were measured using* photometric* assays and an Abbott Architect C16000 analyzer (Abbott Diagnostics, USA). C-reactive protein (CRP) levels were measured via the nephelometric method of the Coulter Image 800 device (Beckman, USA). Hematological tests were performed using the Abbott Cell-Dyn Ruby analyzer (Abbott Diagnostics, USA). Coagulation parameters were measured via a Diagnostica Stago kit in an STA Compact Coagulation analyzer (MA, USA).

#### 2.2.1. NGAL

The concentration of NGAL was measured using the enzyme-linked immunosorbent assay (ELISA) method. We used a commercially available human NGAL ELISA kit (MyBiosource, USA). The procedure for the usage of the ELISA method was performed according to the instructions provided by the manufacturer. The absorbance was measured at a wavelength of 450 *η*m using the ELISA reader. The levels of NGAL were presented as ng/mL, and the sensitivity for the NGAL assay was <10 pg/mL.

### 2.3. Statistical Analysis

A data analysis was performed using SPSS for Windows (ver. 13.1; SPSS, Chicago, IL, USA). All the results were analyzed by the Kolmogorov Smirnov test for the determination of the normal and nonnormal data distributions. The statistical significance of the differences in all parameters between the CCHF and control groups was analyzed using independent sample *t*-tests for normal distribution parameters and the Mann-Whitney *U* test for nonnormal distribution parameters. The relationship between the variables was analyzed with Pearson's correlation. Stepwise multivariate (MVA) logistic regression analyses were performed for the same data, as well. Results were given as odds ratio (OR), and 95% confidence interval (Cl), and the differences were considered significant at *P* < 0.05.

## 3. Results

White blood cell, neutrophil, lymphocyte, and platelet counts of the CCHF patients were significantly lower than of the control group. The prothrombin time and international normalized ratio (INR) of the CCHF patients were significantly higher than of the control group. The demographic characteristics and the hematological parameters of the patients are shown in Table 1.

The NGAL, AST, ALT, creatinine, total bilirubin, indirect bilirubin, lactate dehydrogenase, creatine phosphokinase, and CRP levels of the CCHF patients were significantly higher than those of the control group. All the biochemical parameters are shown in Table 2.

A Pearson correlation analysis showed the RT to be positively correlated with the serum NGAL, CRP, AST, ALT, creatinine, prothrombin time, and INR levels. The RT was negatively correlated with the white blood cell, neutrophil, and platelet counts. Serum NGAL level was negatively correlated with the neutrophil numbers. All the correlation analysis results are shown in Table 3.

An MVA stepwise logistic regression analysis showed the increased levels of serum NGAL (OR 0.3, 95% Cl 0.1–0.4, *P* < 0.001), low platelet counts (OR 0.1, 95% Cl 0.11–0.2, *P* < 0.001), prolonged prothrombin time (OR 0.9, 95% Cl 0.5–1.3, *P* < 0.001), and INR (OR 11.7, 95% Cl 9.0–14.3, *P* < 0.001) to be independent prognostic factors in predicting the RT. Other parameters are not statistically significant.

## 4. Discussion

According to the results of our study, the white blood cell, neutrophil, lymphocyte, and platelet counts of the patients group were significantly lower than of the control group. During the course of the CCHF disease, the excessive release of proinflammatory cytokines decreases the neutrophil, lymphocyte, and platelet counts by suppressing bone marrow and stimulating hemophagocytosis [16]. In our study, serum NGAL level was found to have a strong inverse correlation with neutrophils, lymphocytes, and platelets. In addition, while the RT of the CCHF patients was negatively correlated with neutrophil, lymphocyte, and platelet levels, it was positively correlated with NGAL. A previous study reported NGAL to be synthesized during an early stage of neutrophil maturation. However, its synthesis stops with the induction of neutrophil maturation [17]. The reason for the elevated NGAL level in our study may be related to the release of young neutrophils from the bone marrow to the peripheral circulation. Due to the correlation of low white blood cell and platelet counts with both RT and NGAL level, they may be considered predictive factors for the estimation of RT. Additionally, MVA has shown NGAL level and platelet count to be strong predictors for RT. An elevated NGAL level may demonstrate long RT in CCHF patients.

According to the results of our study, the AST, ALT, INR, and creatinine levels of the CCHF patients were higher than of the control group. While serum NGAL level was positively correlated with AST and ALT, it had no correlation with creatinine level. RT had a strong correlation with AST, ALT, and creatinine. Both the liver and kidneys are damaged during the course of CCHF due to the excessive release of cytokines. Acute liver and renal failure may occur due to this damage, which increases mortality. NGAL is released from neutrophils, macrophages, renal tubular epithelial cells, and hepatocytes [1]. It is known that serum NGAL level increases with acute renal and liver failure, and it is an important prognostic factor, especially for acute renal failure [18]. In the current study, the strong correlation of serum NGAL level with INR, AST, and ALT suggests it to be a prognostic factor in the estimation of the RT in CCHF patients. In this study, while creatinine level was correlated with RT, it was not correlated with NGAL. It is known that the NGAL level increases in acute renal failure during severe infectious diseases [19]. We might not have found a correlation between NGAL and creatinine due to the normal level of it in our patients.

CRP level is not a good prognostic factor in viral infections [20]. In the current study, the CRP level of the patients was higher than that of the control group. However, CRP was not found to be a prognostic factor for CCHF. The NGAL level might be increased more than CRP secondary to tumor necrosis factor-alpha stimulation in CCHF patients, and we might not have found a correlation between NGAL and CRP due to the insufficient elevation of CRP in many cases. In this study, the levels of lactate dehydrogenase, indirect bilirubin, and creatine phosphokinase of the CCHF patients were found to be higher than of the control group. RT had a strong association with lactate dehydrogenase, indirect bilirubin, and creatine phosphokinase. The NGAL level did not have a strong correlation with these parameters. Lactate dehydrogenase, indirect bilirubin, and creatine phosphokinase levels are increased due to cell and muscle damage secondary to the excessive release of cytokines and high fevers in CCHF patients [21]. Even though the elevations of these parameters show a strong association with RT, MVA has shown that lactate dehydrogenase, indirect bilirubin, and creatine phosphokinase levels are not independent predictors. Contrary to these parameters, an elevated NGAL level indicates a strong association with RT. MVA has shown the NGAL level to be an independent predictive factor for RT estimation in CCHF patients, unlike these parameters.

### 4.1. Conclusion

Serum NGAL level is elevated in response to systemic inflammation during bacterial infections. However, we have not known that it also increases during viral infections until now. Serum NGAL level may increase secondary to cytokine release and tissue damage during CCHF. An elevated NGAL level is inversely proportional to the RT. The NGAL level may be a predictive factor during followup with CCHF patients.

## Figures and Tables

**Table 1:** The main characteristics and hematologic and coagulation parameters for the 2 groups.

	CCHF (mean ± SD)	Control (mean ± SD)	*P* value
Age (year)	42.2 ± 12.8	40.3 ± 10.8	0.486
Gender (M/F) (*n*)	21/19	17/17	0.330
Recovery time (days)	12.8 ± 2.5		
WBC (×10^9^/L)	2.6 ± 1.0	7.5 ± 2.5	0.001
Neutrophils (×10^9^/L)	1.4 ± 0.8	4.7 ± 2.1	0.001
Lymphocytes (×10^9^/L)	0.8 ± 0.4	2.1 ± 0.8	0.001
Hb (g/dL)	13.3 ± 2.0	13.7 ± 1.9	0.210
Platelets (×10^9^/L)	80.9 ± 42.5	257.1 ± 55.9	0.001
PT (sec)	14.8 ± 2.5	13.0 ± 1.0	0.001
aPTT (sec)	32.0 ± 5.5	30.5 ± 3.3	0.151
INR	1.8 ± 0.2	1.3 ± 0.1	0.001

CCHF: Crimean-Congo hemorrhagic fever; M: male; F: female; WBC: white blood cell counts; PT: prothrombin time; aPTT: activated partial thromboplastin time; INR: international normalized ratio.

**Table 2:** Results of biochemical parameters for the CCHF and control groups.

	CCHF	Control	*P* value
NGAL (ng/mL)	310.5 ± 48.9	268.3 ± 41.0	0.001
FPG (mg/dL)	108.3 ± 24.8	97.7 ± 9.4	0.106
AST (IU/L)	162.7 ± 167.1	22.8 ± 9.7	0.001
ALT (IU/L)	98.4 ± 90.1	20.5 ± 13.7	0.001
Urea (mg/dL)	34.9 ± 6.5	34.1 ± 8.0	0.637
Creatinine (mg/dL)	1.0 ± 0.2	0.9 ± 0.2	0.001
Tbil (mg/dL)	1.2 ± 0.5	0.8 ± 0.3	0.001
Ibil (mg/dL)	0.8 ± 0.5	0.3 ± 0.1	0.001
LDH (IU/L)	465.6 ± 265.2	193.0 ± 32.6	0.001
CPK (IU/L)	461.3 ± 545.4	68.9 ± 17.1	0.001
CRP (mg/dL)	2.5 ± 3.1	0.7 ± 0.6	0.028
ESR (mm/hr)	20.4 ± 14.9	16.8 ± 14.1	0.289

CCHF: Crimean-Congo hemorrhagic fever; NGAL: neutrophil gelatinase-associated lipocalin; FPG: fasting plasma glucose; AST: aspartate aminotransferase; ALT: alanine aminotransferase; Tbil: total bilirubin; Ibil: indirect bilirubin; LDH: lactate dehydrogenase; CPK: creatine phosphokinase; CRP: C-reactive protein; ESR: erythrocyte sedimentation rate.

**Table 3:** The correlation between recovery time, NGAL level, and hematological and biochemical parameters in patients with CCHF and control group.

	Recovery time		NGAL	
	*r*	*P*	*r*	*P*
Age	0.102	0.387	0.041	0.729
Gender	0.081	0.494	0.040	0.735
WBC	−0.779	0.001	−0.451	0.001
Neutrophil	−0.774	0.001	−0.372	0.001
Lymphocyte	−0.694	0.001	−0.396	0.001
Hb	0.115	0.329	0.074	0.533
Platelet	−0.851	0.001	−0.413	0.001
PT	0.361	0.002	0.059	0.616
aPTT	0.097	0.412	−0.002	0.989
INR	0.848	0.001	0.311	0.007
FPG	0.285	0.014	0.214	0.068
Urea	0.042	0.720	0.008	0.949
Creatinine	0.347	0.002	0.165	0.161
Tbil	0.316	0.006	0.181	0.123
Ibil	0.494	0.001	0.261	0.025
AST	0.497	0.001	0.312	0.007
ALT	0.523	0.001	0.358	0.002
LDH	0.549	0.001	0.235	0.045
CPK	0.403	0.001	0.134	0.256
CRP	0.371	0.001	0.129	0.273
ESR	0.168	0.154	0.239	0.041
NGAL	0.556	0.001		

CCHF: Crimean-Congo hemorrhagic fever; NGAL: neutrophil gelatinase-associated lipocalin; M: male; F: female; WBC: white blood cell counts; PT: prothrombin time; aPTT: activated partial thromboplastin time; INR: international normalized ratio; FPG: fasting plasma glucose; Tbil: total bilirubin; Ibil: indirect bilirubin; AST: aspartate aminotransferase; ALT: alanine aminotransferase; LDH: lactate dehydrogenase; CPK: creatine phosphokinase; CRP: C-reactive protein; ESR: erythrocyte sedimentation rate.
